# Reconditioned monocytes are immunomodulatory and regulate inflammatory environment in sepsis

**DOI:** 10.1038/s41598-023-42237-4

**Published:** 2023-09-11

**Authors:** Kshama Jain, K. Varsha Mohan, Gargi Roy, Prakriti Sinha, Vignesh Jayaraman, Kiran, Ajit Singh Yadav, Akshay Phasalkar, Deepanshu, Anupa Pokhrel, Nagarajan Perumal, Nitin Sinha, Kiran Chaudhary, Pramod Upadhyay

**Affiliations:** 1 National Institute of Immunology, Aruna Asaf Ali Marg, New Delhi, 110067 India; 2 Department of Medicine, Dr. Ram Mahohar Lohia Hospital, Baba Kharak Singh Road, New Delhi, 110001 India; 3 Department of Transfusion Medicine, Dr. Ram Mahohar Lohia Hospital, Baba Kharak Singh Road, New Delhi, 110001 India

**Keywords:** Immunology, Stem cells, Medical research

## Abstract

Sepsis is caused by dysregulated immune response to severe infection and hyper inflammation plays a central role in worsening the disease. The immunomodulatory properties of mesenchymal stem cells (MSCs) have been evaluated as a therapeutic candidate for sepsis. Reconditioned monocytes (RM), generated from healthy human peripheral blood mononuclear cells (PBMCs) exhibit both macrophage and MSCs-like properties. RM were administered at different stages of sepsis in a mouse model. It reduced serum levels of IL6, MCP-1, IL-10, improved hypothermia, increased survival, and recovery from 0 to 66% when combined with antibiotics in the mouse model. The reduced human leucocyte antigen DR molecules expression on RM enables their co-culture with PBMCs of sepsis patients which resulted in reduced ROS production, and up-regulated TGF-β while down-regulating IL6, IL8, and IL-10 in-vitro. RM are potentially immunomodulatory, enhance survival in sepsis mouse model and modulate inflammatory behaviour of sepsis patient’s PBMCs.

## Introduction

Sepsis is the unregulated systemic immune response to infection, resulting in symptoms of physiologic, pathologic, biochemical abnormalities and organ dysfunction^1^. It is a common condition in hospitals and one of the most common causes of mortalities in ICUs. It is estimated to have a global burden of more than 30 million people every year, potentially leading to around 6 million deaths^2^.

Classical immune response against any bacterial or viral infection allows the immune system to clear the infectious agent without disturbing the systemic homeostasis. In severe bacterial infections such as in sepsis the immune homeostasis between inflammatory and anti-inflammatory is compromised causing severe damage to organs while trying to clear the heightened infection^3^. This condition leads to impaired interconnections between innate immune pathways and cytokines production, as well as dampens body’s ability to counter invading pathogens^4,5^. Studies on sepsis indicates that the dysregulation not only damages the system’s disease neutralizing response by the mature cells but also permanently dampens the bone marrow’s hematopoietic stem cells reserves^4^. Most of the studies conducted on sepsis have highlighted the central role of inflammation in worsening the disease conditions.

Numerous strategies focusing on lowering the inflammatory milieu in the body have been extensively investigated as a means of treating acute hyperinflammatory disorders and the resulting organ damage. However, most of these approaches proved inefficient in managing the disease in clinical scenario^6^.

In order to normalize the dysregulated immune response during sepsis, MSCs are considered promising cell-based therapeutics due to their ability to modulate the immune response^7^. Although the exact mechanisms involved in the immunomodulatory activity of MSCs are not well understood, it appears to be a combination of their ability to regulate broad range of immune cells^8^, and they get activated by inflammatory mediators secreted from activated immune cells^9^. A few pre-clinical studies highlight the potential benefits of cell-based therapies in sepsis management^10^.

Reconditioned monocytes (RM) are cells of myeloid linage generated from peripheral blood derived monocytes^11^. These cells are multipotent in nature^11,12^ and typically these are expected to behave similarly to MSCs in terms of their immunomodulatory properties.

In this study we investigated the immunological properties of RM and its potential effectiveness in addressing the dysregulated immune response commonly observed in sepsis.

## Results

In the first part of the study the phenotypic and functional properties of RM were investigated. Based on the findings of in-vitro experiments, in subsequent parts, the RM cells were used to assess their ability to manage sepsis in-vivo and ex-vivo.

### Functional properties of RM

**Figure 1:**
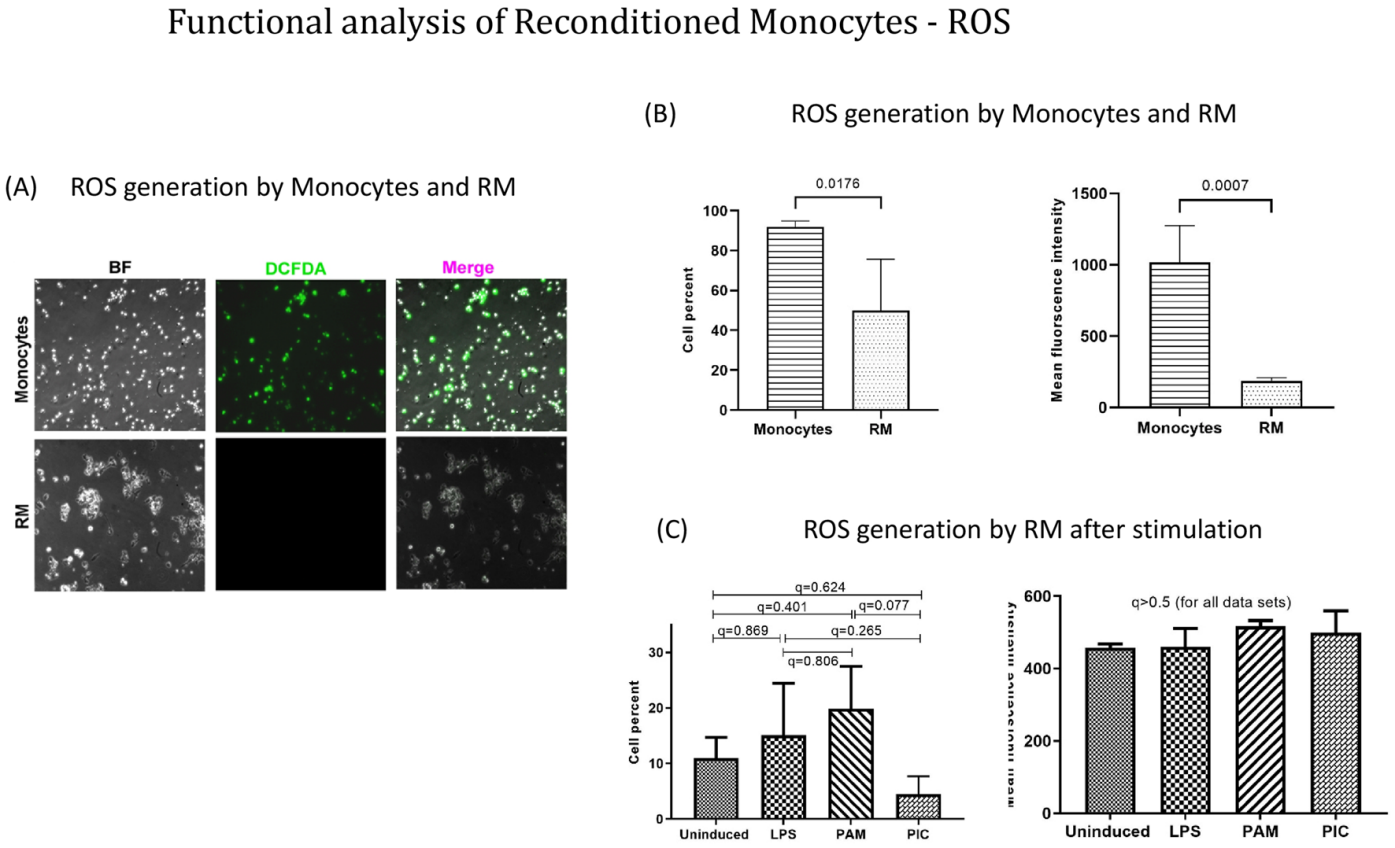
Functional analysis of reconditioned monocytes—reactive oxygen species. (**A**) Representative fluorescent microscope image from one experiment showing ROS generation by monocytes and RM as indicated by green color DCFDA dye. (**B**) Analysis of ROS generation: bar graphs indicating reactive oxidative species (ROS) production by monocytes and RM. Each cell type was incubated for 30 min with DCFDA and ROS production was measured by flow cytometry (n = 4). (**C**) Analysis of ROS generation after stimulation: bar graphs indicating reactive oxidative species (ROS) generation after stimulation of RM with LPS, Poly: IC and PAM3CSK4 for 12 h. Cells were subsequently incubated with DCFDA for 30 min and ROS production was quantified by flow cytometry (n = 4). Results are presented as mean with SD.

To investigate the immunological properties of RM, their inflammatory behavior was assessed by analyzing the generation of Reactive oxygen species (ROS) in un-stimulated and stimulated (LPS, Poly:IC and PAM3CSK4) state using DCFDA dye. ROS production was visualized through fluorescence microscopy (Fig. 1A) and was further quantified using flowcytometry. The analysis revealed significant reduction in percent of ROS producing cells in RM cultures (Fig. 1B). Additionally, the MFI values showed reduction of ROS production by ~ 5.4 folds compared to un-stimulated RM culture (Fig. 1B). The stimulation of RM with TLR agonists LPS, Poly:IC and PAM3CSK4 did not alter the ROS levels in culture (Fig. 1C).

**Figure 2:**
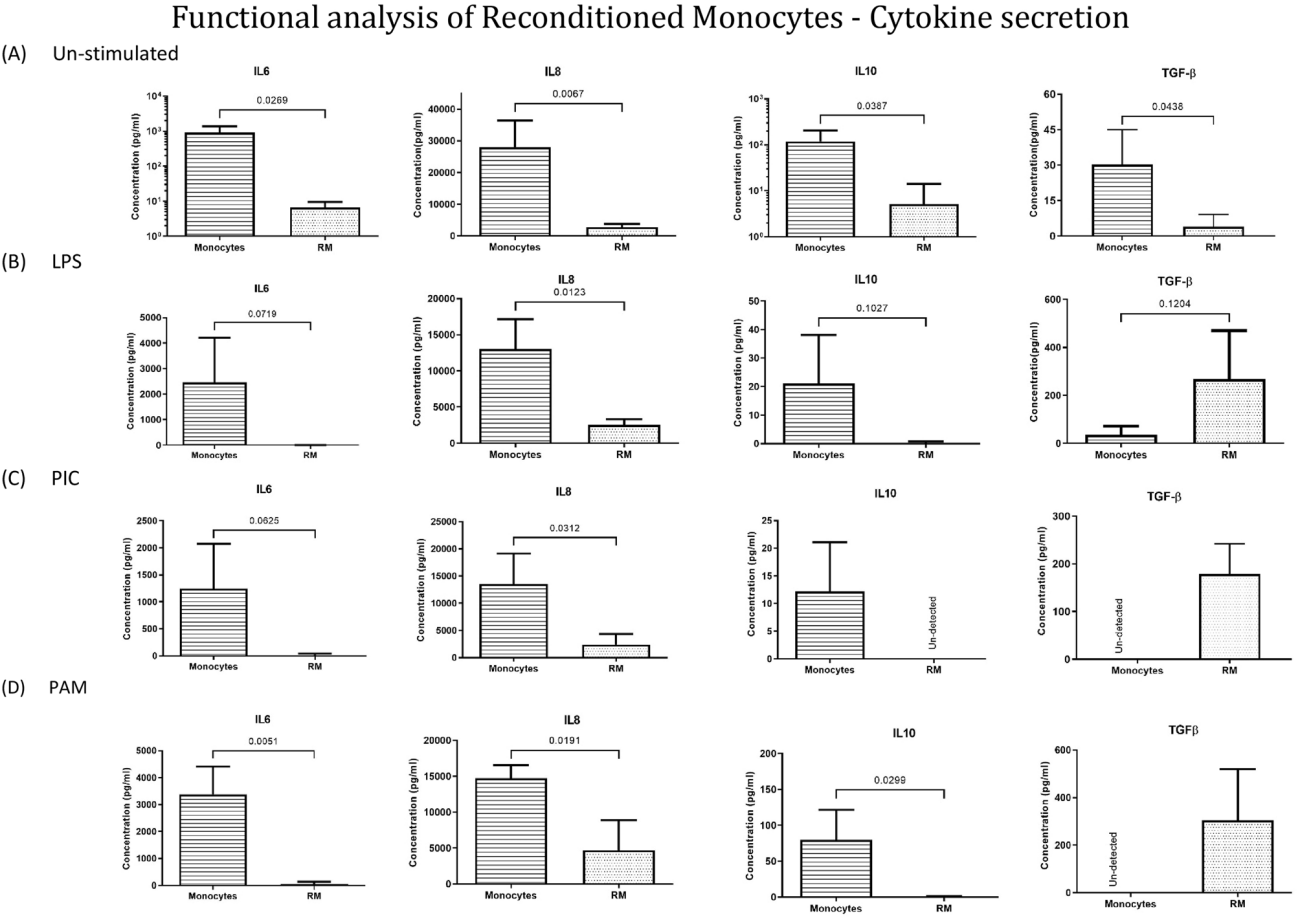
Functional analysis of reconditioned monocytes—cytokine secretion. (**A**) Bar graphs indicating mean concentration of IL6, IL10, IL1β, and IL8 secretion by unstimulated Monocytes and RM (n = 3), (**B**) bar graphs indicating mean concentration of IL6, IL10, IL1β, and IL8 secretion by monocytes and RM after stimulated with LPS for 12 h (n = 3). (**C**) stimulation with Poly:IC for 12 h (n = 3) and (**D**) stimulation with PAM3CSK4 for 12 h (n = 3). Cytokines present in cell culture supernatants were quantified by cytometric bead assay. Results are presented as mean with SD.

The cytokine secretory profile of cells was studied in-vitro in un-stimulated and stimulated state (Fig. 2A–D). The comparative analysis of cytokine secretory profile of un-stimulated RM showed a significantly reduced secretion of pro-inflammatory (IL6, IL8) as well as anti-inflammatory cytokines (IL10) relative to the corresponding monocytes.

### Immunogenicity of RM

**Figure 3:**
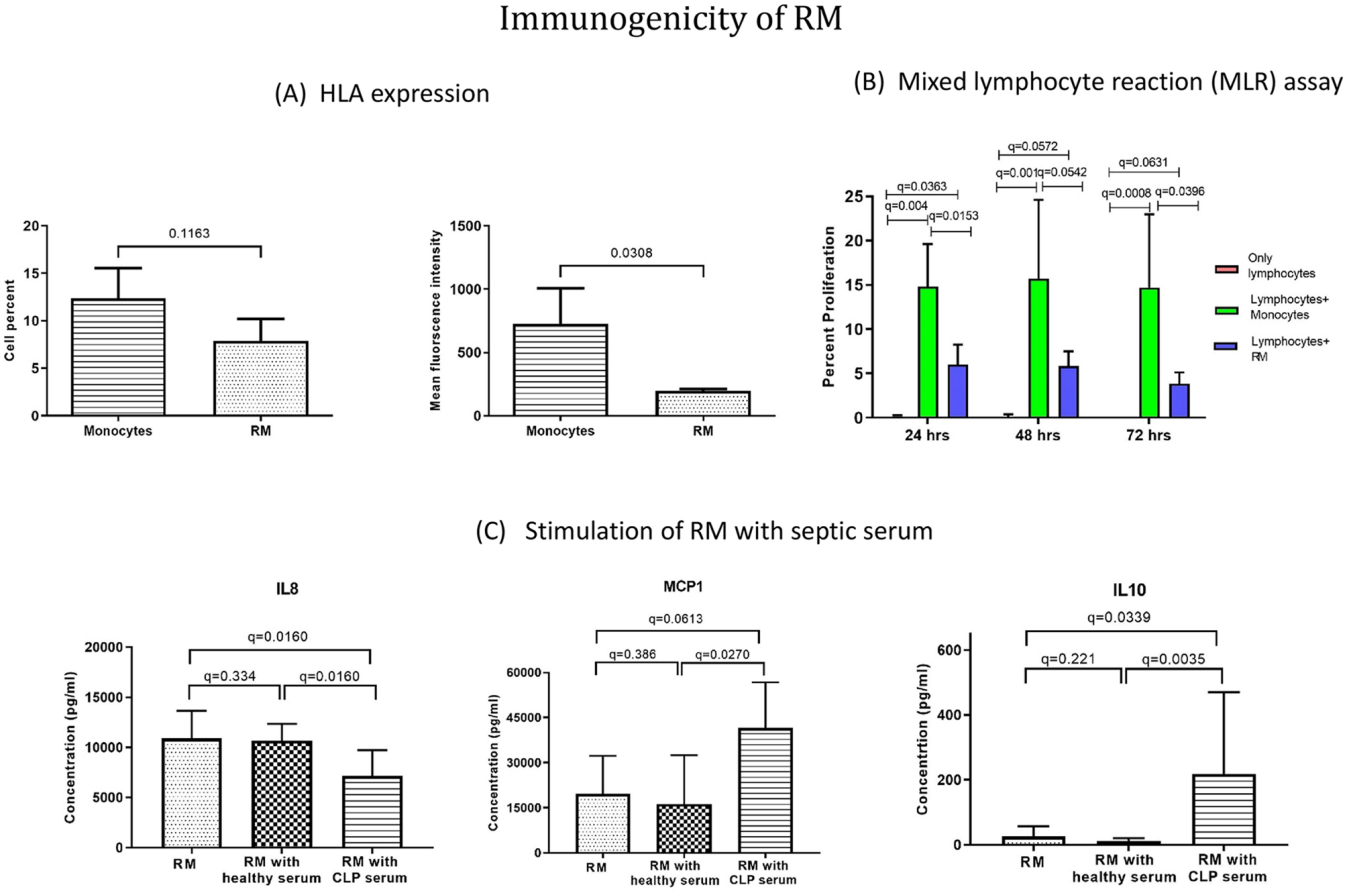
Immunogenicity of RM. (**A**) Evaluation of HLA-DR expression on RM by flowcytometry (n = 3). (**B**) Mixed lymphocyte reaction for assessing immunogenicity of allogenic RM, diagrammatic representation showing experimental details of Mixed lymphocyte reaction, Bar graphs representing percentage of proliferating lymphocytes (responders) in response to allogenic monocyte and RM (stimulator) (n = 6). (**C**) Flow cytometric analysis comparing cytokine secretion by RM, cultured in healthy and septic mice serum. Bar graphs comparing IL8, MCP1 and IL10 secretion by unstimulated, healthy serum stimulated and septic serum stimulated RM (n = 6). Results are presented as mean with SD.

To understand the immunogenic nature of RM, the expression of the key immune antigen HLA-DR was determined by flow cytometry. The analysis showed that 7.88% of cells in RM cultures expressed HLA-DR antigen with an MFI of 195.3 compared to 12.4% of cells, MFI = 725.3, in monocytes; indicating a significant reduction HLA-DR molecules expression (Fig. 3A). The reduced immunogenicity of RM was further confirmed by evaluating their potential to induce lymphocyte proliferation in an in-vitro MLR experiment. The analysis showed that PBMC proliferation induced by RM (6.02%) was significantly lower as compared to PBMC proliferation induced by corresponding monocytes (14.8%). Furthermore, the time kinetic analysis at 24 h, 48 h and 72 h of MLR culture did not show any significant change in lymphocyte proliferation (Fig. 3B).

In order to rule out any deleterious effect of inflammatory milieu on the RM, the serum isolated from normal and mice with sepsis, i.e., 6 h. post CLP, were co-cultured with RM and results are shown in Fig. 3C. Under these conditions, the immunomodulatory property of RM was exhibited and higher levels of IL10 and lesser IL8 were detected, compared to the co-culture of RM and ‘healthy serum’.

As the characterization studies of RM suggested the anti-inflammatory/ immune- modulatory properties of RM, we further investigated the potential of RM in attenuating the systemic inflammation during sepsis.

### RM transplantation in the mouse model of sepsis

**Figure 4:**
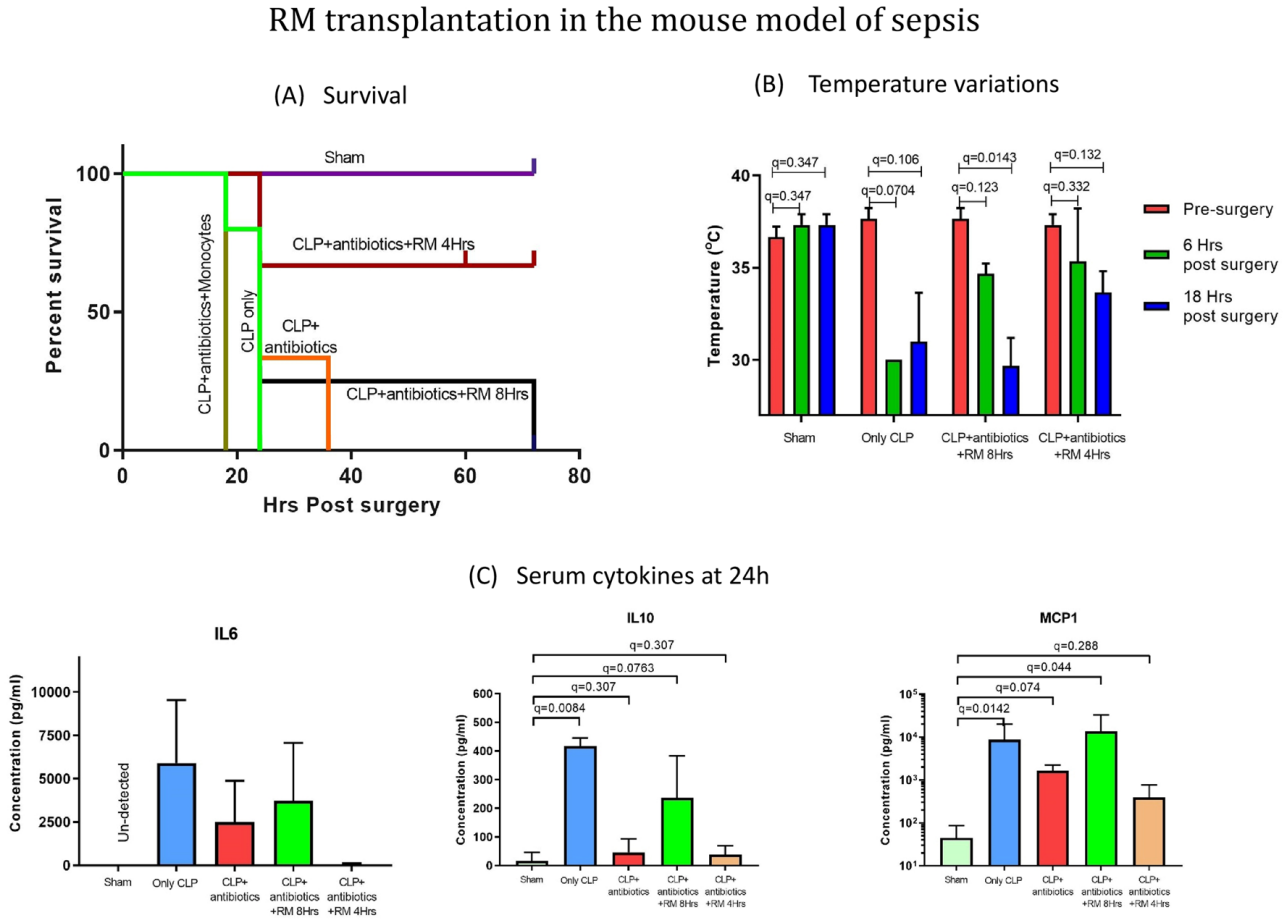
RM transplantation in the mouse model of sepsis. (**A**) Survival rates of mice with CLP-induced sepsis following RM treatment shown as Kaplan–Meier survival curve. (**B**) Temperature changes in mice with CLP-induced sepsis following RM treatment. Surface body temperatures were measured at different time points in the Sham group, CLP group, and different treatment groups. Treatment with RM 4 h post-surgery attenuated temperature decline at 6 h and 18 h post-CLP (n = 3). (**C**) Bar graphs depicting mean concentrations of cytokines in serum of CLP mice and different treatment groups. Serum levels of IL6, IL10 and MCP1 were quantified at experiment termination (22 ± 2 h) in different experimental groups (n = 3). Results are presented as mean with SD.

We investigated the effects of RM on survival of CLP-sepsis BALB/cj mice (Fig. 4). In the control (CLP and CLP + antibiotic) groups, mortality reached 100% at 24 h and 36 h respectively, but in the RM-treated group (CLP + antibiotic + RM after 4 h) 66.6% animals exhibited survival and recovery from sepsis (Fig. 4A). There was no significant improvement in animal survival observed in RM transplantation group where RM was transplanted after 8 h of CLP surgery (Fig. 4A).

#### The systemic inflammation in CLP-sepsis model upon RM transplantation

CLP—sepsis induced an acute increase in both inflammatory (IL-6 and MCP1) and anti-inflammatory (IL-10) serum cytokine concentrations. The administration of RM after 4 h of CLP resulted in improvement in hypothermia, however no improvement in body temperature was observed in the control (CLP only) and 8 h transplantation group (Fig. 4B). The administration of RM 4 h post CLP resulted in reduced serum concentrations of IL 6, MCP-1 and IL-10 at 22 ± 2 h (Fig. 4C).

### Patient recruitment

**Figure 5:**
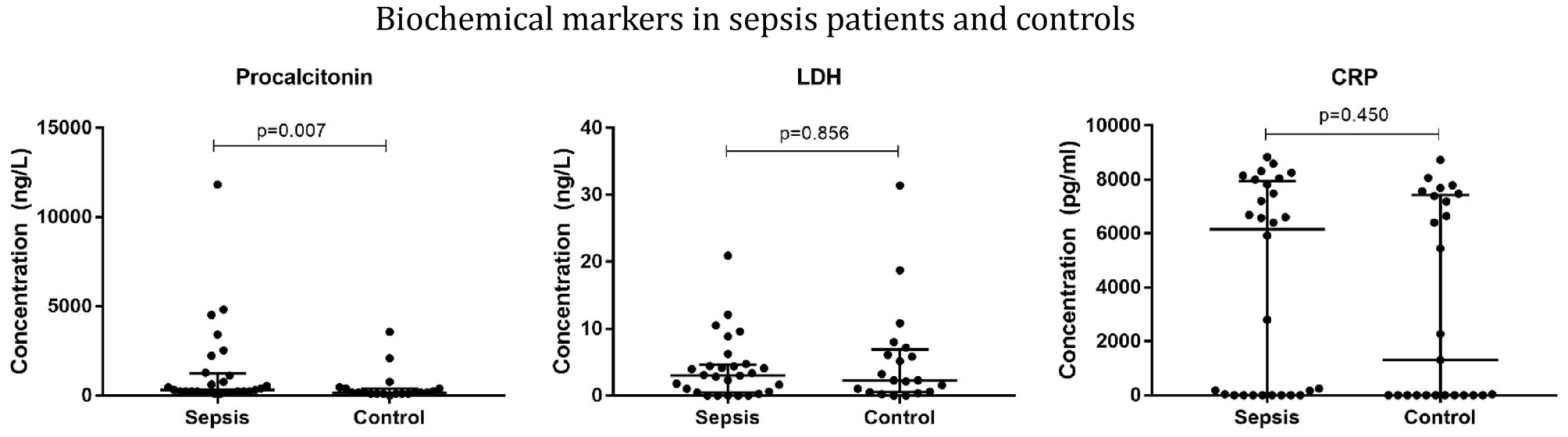
Biochemical markers in suspected sepsis patient plasma (for patients N = 28, for healthy volunteers N = 20). Results are presented as median with inter-quartile range.

A summary of the inclusion and exclusion criteria of sepsis patients is shown in Supplementary Material, Table ST1 and their biochemical parameters are depicted in Fig. 5.

### RM co-culture with suspected sepsis patient derived PBMCs

To evaluate the immune-modulating potential of RM in sepsis, a co-culture of RM and patient derived PBMCs was studied. The RM culture were initiated 6 days prior to the collection of sepsis blood sample and a transwell co-culture of RM with patient PBMCs were setup.

#### The inflammatory behavior of host PBMCs

**Figure 6:**
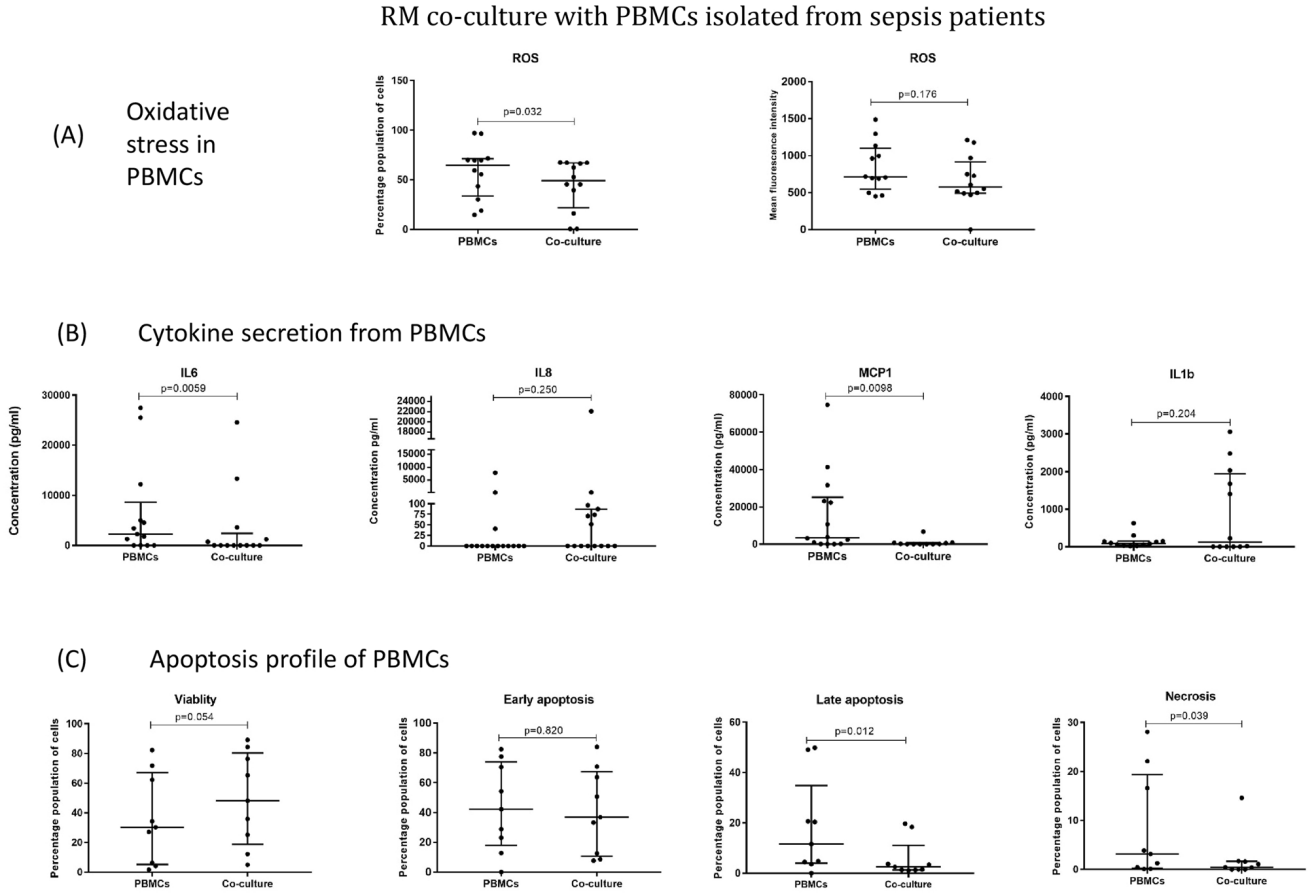
RM co-culture with PBMCs isolated from suspected sepsis patients. (**A**) Evaluation of oxidative stress in patient PBMCs in coculture with RM: Bar graphs showing the percent of cells producing ROS and the extent of ROS generation by cells in PBMCs cultures and PBMC-RM cocultures as quantified by DCFDA dye (N = 12). (**B**) Changes in cytokine secretory profile of cells in PBMCs cultures and PBMC-RM cocultures quantified by cytometric bead assay (N = 15). (**C**) Evaluation of apoptosis profile of patient PBMCs after coculture with RM: bar graphs showing the percent of viable cells and percent of cells in various phases of apoptosis in PBMCs cultures and PBMC-RM cocultures Quantified by Annexin-V/PI staining (N = 9). Results are presented as median with inter-quartile range.

The ROS levels by patient PBMCs was evaluated after their 24 h co-culture with RM. The flow cytometric analysis showed that RM induced reduction in ROS production by patient PBMCs. The number of PBMCs generating ROS as well the ROS concentration both showed reduction in cocultures (Fig. 6A).

The reduced inflammatory behavior of patient PBMCs was further checked by quantifying the concentration of pro-inflammatory cytokines in the 24 h co-culture supernatants by cytometric bead array. CBA analysis of coculture supernatants showed a trend of reduced levels of IL6, IL8, MCP1 and IL1β (Fig. 6B).

#### The apoptosis profile and viable cells of host PBMCs

The Apoptotic profiling of patient derived PBMCs co-culture of RM, was done by Annexin V staining and followed by flow cytometric analysis. The apoptosis profiling showed a trend of increase in percent of Annexin-V^-^ PI^-^ viable cells and reduction in AnnexinV^+^ PI^+^ necrotic cell population in RM-PBMC cocultures (Fig. 6C).

## Discussion

This study revolves around Reconditioned Monocytes generated from the monocytes isolated from healthy peripheral blood. In this study, first we established that RM have immunomodulatory properties and an attenuated immunogenicity. These unique features of RM were exploited to normalize the uncontrolled immune environment seen in sepsis. The success of RM transplantation in mitigating systemic inflammation and improving survival was confirmed in a cecal ligation and puncture (CLP) polymicrobial sepsis mouse model of sepsis. Finally, we demonstrated the immune-modulating potential of RM on PBMCs isolated from suspected sepsis patients.

Since the precursor cells of RM are cells of immune origin and RM are known to express reduced levels of monocytic markers^13–16^, it becomes vital to understand the effect of reconditioning on the extent and type of changes induced in the immune properties of RM.

We have earlier reported^11^ that healthy peripheral blood derived monocytes were used as precursor cells for generation of RM^17–20^. During the reconditioning culture non-monocytic cells are eliminated and a morphologically uniform cell population is achieved (Supplementary Material, Fig. S1B). The spindle shape of RM indicated their morphological similarity with mesenchymal stem cells^21^ as well as alternative macrophages^22^.

The role of reactive oxygen species (ROS) in regulating stem cell dynamics is well established^23,24^ and low ROS is essential for their maintenance and proliferation^25^. Low ROS in RM cultures both in uninduced as well as TLR1/2, TLR3, TLR4 induced condition indicates polarization of RM from inflammatory to a non-inflammatory state. Cytokine secretion by immune cells is an important process to mediate their immunological functions^26^. Under stimulation the reduced secretion of proinflammatory cytokines by RM, in comparison to their corresponding stimulated monocytes, confirmed a shift in the immunological properties of RM.

The diminished levels of IL10 in our study, which is typically known as an anti-inflammatory cytokine, is not necessarily a contradiction as reports suggest that MSCs secrete IL-10 only under an inflammatory environment in the presences of cytokines such as TNFα, IFNγ and IL-1b^27^. This is perhaps the reason behind the contradicting reports on the secretion profile of IL-10 by MSCs. While some studies report higher secretion of IL-10 by MSCs^28–31^, there are a few who discards such a possibility^32^. In our data too, while diminished IL-10 was observed in in-vitro conditions (Fig. 2A–D), higher levels were detected when RM were activated with the serum of mice in sepsis (Fig. 3C).

To determine the possibility of allogenic transplantation of RM across MHC mismatched cases, the immunogenicity of RM was analysed by phenotyping the cells for HLA-DR expression. The flowcytometric analysis of RM and monocytes depicted significant reduction in HLA-DR expression in RM as compared to corresponding monocytes (Fig. 3A). This reduction in MHC-II expression suggested reduction in immunogenicity of RM. The low immunogenicity of RM was further validated by determining its potential to induce proliferation of HLA mismatched allogenic T cells in in-vitro mixed lymphocyte reaction (Fig. 3B).

In order to analyse the ability of RM to modulate inflammatory environment a sepsis model was generated by performing the CLP surgery (Supplementary Material, Section II) in BALB/cj mice.

While humanized mice may seem like an appropriate model, this has many limitations^33–35^. Since our focus was towards the primary evaluation of cell-based therapeutics for rescue in sepsis condition, hence a normal mouse model of sepsis was adequate for this objective.

So far, most reports on cell-based therapy have utilized MSCs as the potential candidate for sepsis management^36,37^. However, these studies vary widely in terms of the source of MSCs, number of cells transplanted, time of transplantation, route of transplantation and severity of the disease. Since RM also demonstrate immunomodulatory behaviour, the parameter for RM transplantation like number of cells transplanted and site of transplantation were standardized based on the available literature of MSCs transplantation^38,39^. The time of transplantation was standardized according to the systemic inflammatory response of our animal model. As the inflammatory environment in our sepsis model peaked at 6 h post-surgery, (Fig. S3H), the cell transplantation time was chosen before (i.e., 4 h post-surgery) and after (i.e., 8 h post-surgery) the inflammation maxima.

The effectiveness of the therapy in RM treated and untreated groups was determined by comparing the survival curves. The survival data from control and experimental groups showed a significantly reduced mortality in animals which received a treatment of RM along with antibiotic at 4-h post-surgery, indicating effectiveness of RM in improving sepsis associated mortality (Fig. 4A). However, RM transplantation at 8-h post-surgery failed to improve the animal survival. This time dependent effective-ness of RM transplantation indicated the importance of a timely immunomodulatory intervention as provided by the RM in our treatment plan. The ineffectiveness of only antibiotic treatment in reducing absolute animal mortality further established the efficacy of RM in sepsis management. Furthermore, the positive effect of RM therapy in 4-h transplantation group was also shown by their better ability to regulate the body temperature of the animals as compared to other experimental groups (Fig. 4B).

Additionally, the analysis of serum cytokine levels in moribund stage showed that the 4-h RM transplantation group was able to regulate the cytokine levels almost like the sham control thereby confirming the role of immunomodulatory effect of RM in managing sepsis (Fig. 4C).

In the next step, we explored the possible utility of RM in managing sepsis in human patients. Patients with critical signs infection having qSOFA score ≥ 2^1^ and a few healthy controls were enrolled and their relevant biochemical parameters were estimated.

As commonly expected, significantly higher pro-calcitonin levels were found in suspected sepsis patients than the control^40^. We observed lower creatinine levels in sepsis patients (Supplementary Material, Fig. S4), which was typically unexpected. Although sepsis is known to reduce the production of creatinine which limits the net increase in serum creatinine after sepsis^41^, the observed creatinine levels was more like an anomaly. There was not much difference observed in lactate dehydrogenase (LDH) and C-reactive protein (CRP) levels.

In the ex-vivo evaluation of RM, we cultured RM with the PBMCs isolated from suspected sepsis patients. Like the findings in the animal model, the RM was able to weaken the inflammatory condition of PBMCs as reflected by the decreased ROS generation in RM co-culture group (Fig. 6A). Similar pattern was reflected in the cytokine levels of IL6 and MCP1 (Fig. 6B).

In this experiment, as a possible consequence of attenuated inflammatory condition of PBMCs, a significantly increased viability of cells and slightly fewer early apoptotic PBMCs of the host was observed (Fig. 6C).

Apoptosis is a tightly regulated reaction and during acute sepsis, primarily the excessive apoptosis of immune cells leads to immunosuppression^42^. In the prolonged immunosuppressive state, patients often succumb to opportunistic secondary infections^43^. The therapeutic possibilities of controlling sepsis-induced immune cell apoptosis have been recently suggested^44^. It is of interest to emphasize that induction of RM was able to increase the viability of immune cells (PBMCs) and it can delay the onset of immunosuppressive state in sepsis^45^.

There are a few limitations of this study.

Among the healthy controls, though everyone had a normal and healthy daily routine, some participants presented with a few atypical parameters and secondly, the median age of healthy controls was 28 years, whereas for suspected sepsis patients it was 50 years.

While recruiting patients for this study, almost all recruited patients have had some antibiotics prior to our blood sampling. In managing sepsis, it is typically believed that each hour’s delay in initiating antibiotics costs lives. Although some caution is suggested^46^, it is a generally accepted approach to begin antibiotics administration without any delay^47^. Perhaps this was the reason for inconsistencies in some of the biochemical parameters, like LDH and CRP. Also due to this limitation, we could not exactly identify the immunological status of patients. This was important as in the mice study, RM transplantation showed promising recovery when they were administered just before the onset of severe sepsis (Fig. 4A). Secondly, since the focus of this study was on evaluating the RM for modulating hyper-inflammatory conditions in sepsis, we focussed on patients with infection and qSOFA Scores ≥ 2. We did not classified patients based on severity, presence of organ support, pathogen, or the site of infection, and these parameters may also influence the magnitude of the inflammatory/dysregulated host response to affect the observed outcome.

In this study several comparisons among multiple groups with small sample size have been done and in most of the comparisons, the corrections for multiple comparisons have been done. These comparisons result in limited conclusions discussed above and may serve to generate broader hypotheses.

In summary, the RM is a unique cell type having immunomodulatory ability. In the mouse model of sepsis, the transplantation of RM in initial phase of disease tends to regulate the systemic inflammatory response of the immune system. The RM co-culture with the PBMCs of suspected sepsis patients provides a proof of the concept as it showed similar trend of reducing the inflammatory environment which can positively influence the outcome.

The easy availability of healthy ‘buffy coat’ from blood bank and a straightforward reconditing protocol makes RM a promising therapeutic candidate for regulating immune-inflammatory environment in sepsis.

## Methods

### Ethics statement

The investigation on mice was approved by the Institutional Animal Ethics Committee (IAEC# 479/18) of National Institute of Immunology (NII), New Delhi.

All animal experiments were performed in accordance with the guidelines on the regulation of Committee for Control and Supervision of Experiments on Animals, Ministry of Fisheries, Animal Husbandry and Dairying, Government of India, under the supervision of a professional Veterinarian at the Small Animal Facility of NII, New Delhi. All animal experiments and reporting also adhere to the ARRIVE guidelines.

Blood samples from suspected sepsis patients were obtained from the Department of Medicine, Dr. Ram Manohar Lohia (RML) Hospital, New Delhi, after obtaining written informed consent from the patients. The recruitment of healthy volunteers and all the experimental work was carried out at NII. The investigation was approved by the Institutional Human Ethics Committee of Dr. RML Hospital (File No. 474(10/2021)IEC/ABVIMS/RMLH/493) and the Institutional Human Ethics Committee (IHEC#118/19) of NII.

All experiments were performed in accordance with National Ethical Guidelines for Biomedical and Health Research Involving Human Participants issued by the Indian Council of Medical Research, India.

### Study design

#### In-vitro study

For in-vitro cell-based experiments, PBMCs were first isolated from blood through density-based separation followed by isolation of monocytes from PBMCs based on their property to adhere to plastic tissue culture plates. The monocytes were then cultured in a defined media for 6 days to allow development of the Reconditioned Monocytes (RM).

To assess the immunomodulatory behaviour, the RM were analysed for their ROS generation ability and cytokine secretion profile in unstimulated and stimulated state. Cells were analysed for generation of Reactive Oxygen Species (ROS) by DCFDA assay. 2’,7’-Dichlorodihydrofluorescein diacetate (H2-DCFDA) is a cell permeable dye which can diffuse into the live cells. Inside the cell it is deacetylated by cellular esterases to a non-fluorescent compound. The deacetylated compound is oxidized by ROS into 2’,7’-dichlorofluorescein (DCF) which is highly fluorescent and detected by flow cytometry.

Cytokine secretory profile of cells was investigated on activation by different TLR stimulants, PAM3SK4 ligand for TLR1/2), polyinosinic-polycytidylic acid or poly(I:C) for TLR3 or LPS for TLR4. Levels of secreted cytokines was analysed by Cytometric Bead Array (CBA). Further the immunogenicity of RM in allogenic setup was determined by MLR (Mixed Lymphocyte Reaction) reaction.

#### Animal study

To understand the protective effect of RM in sepsis, a mouse model of sepsis was generated by performing ceacal ligation and puncture (CLP) surgery in animals. The severity of sepsis after CLP surgery was validated by analyzing various physical (body temperature), behavioral (animal movements and feeding patterns), biochemical (serum glucose concentration, liver damage markers and serum cytokine levels) parameters and the mortality of animals (Supplementary Material Fig. S3).

The animals in different experimental groups were administered with the corresponding treatments (Table 1) and were analyzed for protection against sepsis based on their morbidity and mortality after treatment in each experimental group. To understand the role of RM in protection against sepsis, the serum cytokine profile of animals was studied using CBA at 22 ± 2 h of surgery. For all the biochemical assays blood/serum samples were collected at the time of death or 24 h post-surgery (whichever occurred earlier).

**Table 1:** Time line for CLP induced sepsis and treatment in mice.

Pre-surgery			Post-surgery		
Group	N^a^	0 h	4 h	8 h	24 h
		Procedure	Injection	Injection	Procedure
Sham	6	Sham surgery	Saline		Termination
Only CLP	6	CLP surgery	Saline		Termination
CLP + antibiotics	4	CLP surgery	Antibiotics		Termination
CLP + antibiotics + monocytes 4 h	6	CLP surgery	Monocytes + antibiotics		Termination
CLP + antibiotics + RM 4 h	6	CLP surgery	RM + antibiotics		Termination
CLP + antibiotics + RM 8 h	4	CLP surgery	Antibiotics	RM + antibiotics	Termination

^a^Number of animals in the group.

#### Patient samples

For deciphering the effect of RM on septic PBMCs, peripheral blood samples were collected from patients admitted in the Departments of Medicine, Ram Manohar Lohia Hospital. Patients with clinical sign of infection and written, informed consent were eligible for participation in the study. Only patients which were given a qSOFA scoring of ≥ 2 were characterized as septic suspect and included. Further, patients with age < 18 years/any malignancy/chronic inflammatory diseases/traumatic brain injury or HBV/HCV/HIV infection were excluded.

The details of participation, inclusion and exclusion criteria is also given in Supplementary Material, Table ST1. Around 8–10 ml blood sample thus collected was tested for HBV/HCV/HIV infection using rapid antigen kits (J. Mitra & Co. Pvt. Ltd. India). Samples with any of the infection were rejected.

Twenty-eight suspected sepsis patient samples (13 M/15 F, median age 50 years) were collected and processed. Twenty healthy controls were recruited at NII (11 M/9 F, median age 28 years).

#### Processing and study

Serum concentrations of sepsis-related biochemical markers creatinine, procalcitonin and C-reactive protein were tested in all the samples.

For RM generation, buffy coat bags were collected from the Blood Centre, Department of Transfusion Medicine, Dr. RML Hospital.

For setting up trans-well co-culture of patient derived PBMCs with healthy RM, PBMCs were isolated by density-based centrifugation and were seeded in 12 well plates. RM were seeded in transwells placed over the PBMC plated wells. The ratio of PBMCs and RM seeding density was maintained at 4:1 respectively. After 24 h of coculture, culture supernatants were collected for cytokine analysis and both cell types were collected separately for apoptosis profiling and estimation of ROS generation. The schematic flow of sample processing is depicted in Supplementary Material, Fig. S5.

## Experimental methods

### Reconditioning procedure

For the process of reconditioning, monocytes were cultured in-vitro for a period of 6 days in culture media consisting of Iscove’sModified Dulbecco’s Media (IMDM) as basal media (Gibco, Waltham, MA, USA) supplemented with 4.0 ng/ml interleukin-3 (IL3) (ProSpec, Israel/ImmunoTools GmbH, Germany), 5.0 ng/ml Macrophage Colony Stimulating Factor (MCSF)(ProSpec, Israel/ ImmunoTools GmbH, Germany), 140 µM β-mercaptoethanol (2-ME or β ME) and 0.5% human Embryonic Stem Cell grade Fetal Bovine Serum (ESC-FBS). A detailed method of RM generation has been published earlier^11,12^.

### RM transplantation

A CLP mouse model of sepsis was created (Supplementary Material, Section II) and the details of time line for CLP induced sepsis and treatment are given in Table 1. A CLP mouse model of sepsis was created (Supplementary Material, Section II). In the treatment group a few combinations of 10^6^ RM, 10^6^ monocytes, antibiotic Ceftriaxone (Cipla, India) (20 mg/kg) in 150 μl of PBS were administered intraperitoneally, either 4 or 8 h after CLP procedure. In the control group, only 150 μl PBS was administered.

### Flow cytometric analysis

For flowcytometric analysis of samples, cells were stained with corresponding fluorescent tagged antibodies as per the guided protocol. The stained samples were run in FACS verse (BD Bioscience, CA, USA). Unstained samples were used for voltage settings of the machine. Color compensation of multiple fluorochromes was done using corresponding single-color controls. The analysis of flowcytometric result was done using FlowJo software (Tree Star, Ashland, OR, USA).

After setting up the gate of positive staining using specific antibody and isotype controls the percentage of stained cells and the average mean fluorescence intensity (MFI) on fluorescent cells were calculated. The MFI depicts average number of fluorescent molecules on a fluorescent cell.

### Cytokines analysis

Cytokine analysis of culture supernatants as well as mice and human serum samples was done using Cytometric Bead array (CBA) as per the guided protocol (BD biosciences, USA). All the samples along with the standards were run in Flow-cytometer (BD FACS Verse, BD Bioscience, CA, USA). The concentration of each cytokine was determined using FCAP Array v3.0 software (BD Bioscience, USA) as per the guided protocol.

### Mixed lymphocyte reaction

For setting up a mixed lymphocyte reaction, PBMCs from healthy donors were used as responders and RM generated from the peripheral blood monocytes of different individuals were used as stimulators. The responders (PBMCs) were first labelled with CFSE dye and were then cocultured with stimulator cells in ratio of 1:1 for the duration of 24 h, 48 h, and 72 h. After the respective incubation periods, cells from the coculture were analyzed by flowcytometry. The proliferation of cells was estimated by the percentage of CFSE^lo/−^ cells after each incubation period and it was compared with percentage at the beginning of the experiment.

### Statistical analysis

Data was analysed and plotted in GraphPad Prism 7 software.

Wherever two sets of data were compared, the unpaired t test was done and p values are shown in the plot. While comparing more than two sets of data, nonparametric ANOVA (Kruskal–Wallis) test was used. The correction for multiple comparison was made by controlling the false discovery rate and the two-stage step-up method of Benjamini, Krieger and Yekutieli was applied and corrected p-values (q-values) are reported.

Comparison of survival curves was done using Log-rank (Mantel–Cox) test.

### Supplementary Information

Supplementary Information.

## Data Availability

All relevant data is contained within the article. The original contributions presented in the study are included in the article/supplementary material, further inquiries can be directed to the corresponding author/s.
