# Supplementary material for: Reconditioned monocytes are immunomodulatory and regulate inflammatory environment in sepsis
Source: Sci Rep. 2023 Sep 11;13:14977. doi: 10.1038/s41598-023-42237-4 (PMC10495550; doi:10.1038/s41598-023-42237-4)
Supplement: Supplementary file 1 — Supplementary Information. [file 41598_2023_42237_MOESM1_ESM.docx]

Supplementary Material

**Reconditioned monocytes are** **immunomodulatory and regulate inflammatory environment in sepsis**

Kshama Jain^1*^, jainkshama7@gmail.com

K Varsha Mohan^1*^, kvarshamohan@nii.ac.in

Gargi Roy^1^, gargi.roy@nii.ac.in

Prakriti Sinha^1^, prakriti@nii.ac.in

Vignesh Jayaraman^1^, vignesh.jayaraman12@gmail.com

Kiran^2^, doctorkiranyadav@gmail.com

Ajit Singh Yadav^2^, ssajitsingh.yadav@gmail.com

Akshay Phasalkar^2^, akshayphasalkar95@gmail.com

Deepanshu^2^, ydeepanshu997@gmail.com

Anupa Pokhrel^3^, anupa000@yahoo.co.uk

Nagarajan Perumal^1^, nagarajan@nii.ac.in

Nitin Sinha^2^, drnitinsinha26@yahoo.co.in

Kiran Chaudhary^3^, drchaudharyk@yahoo.co.in

Pramod Upadhyay^1^, pkumar@nii.ac.in

1. National Institute of Immunology, Aruna Asaf Ali Marg, New Delhi 110067

2. Department of Medicine, Dr. Ram Mahohar Lohia Hospital, Baba Kharak Singh Road, New Delhi 110001

3. Department of Transfusion Medicine, Dr. Ram Mahohar Lohia Hospital, Baba Kharak Singh Road, New Delhi 110001

* These authors share first authorship

Index Page

Section I : Characterization of RM 2

Section II : Cecal ligation and puncture polymicrobial sepsis model 7

Section III : Participants inclusion and exclusion for the study, Table ST1 10

Section IV : Creatinine levels in sepsis patients and controls, Fig. S4 11

Section V : Schematic flow of sample processing, Fig. S5 11

Section VI : List of primers 12

Section I : Characterization of RM

Peripheral blood derived healthy monocytes were used as precursor cells for generation of RM. The duration of the *in-vitro* culture was 6 days and the components of reconditioning media were MCSF, IL3, β-Mercaptoethanol and 0.5% embryonic stem cell grade serum. Both MCSF and IL3 are hematopoietic growth factor that enhances the proliferation and survival of bone marrow progenitor cells. Studies have also shown that IL3 and MCSF have a synergistic effect on monocyte proliferation. β-ME used in the culture cocktail works as a reducing agent. Studies have shown that β-ME supports the long-term proliferation of leucocytes by enhancing the intracellular GSH levels. The foremost advantage of β-ME is to enhance the sensitivity of cells to the *in-vitro* culture environment, thereby increasing the efficiency of reconditioning. Furthermore, lower serum concentration has been shown to improve the effectiveness of β-ME in cell culture.

*Phenotypic characterization of RM*

At the beginning of culture *i.e.* at day 0 of reconditioning, the culture consisted of monocytes as well as small numbers of lymphocytes. However, at the completion of culture duration *i.e.* at day 6, the culture contained a homogenous cellular population representing RM (Fig. S1B). This observation indicates that reconditioning cocktail specifically affected the monocyte population and other cells types which were initially present at D0 of the culture, started undergoing apoptosis and were subsequently removed from the culture when the culture media was changed. As a result of removal of all non-monocytic cells, the RM culture at day 6 were found more homogenous.

The process of reconditioning induces noteworthy changes in the morphology of cells. While the cells in the day 0 culture were smaller in size and round, the cells at day 6 of culture became larger in size and attained a spindle shaped morphology (Fig. S1B). The spindle shape attained by D6 RM indicated their morphological similarity with mesenchymal stem cells as well as alternative macrophages.

After analyzing the presence of various immune cells such as monocytes, granulocytes, and lymphocytes in D0 and D6 cultures, the percentage of various monocytic subsets were evaluated by CD14 and CD16 based flow cytometric analysis of D0 and D6 cells (Fig. S1C). The peripheral blood typically consists of three different monocyte subsets namely classical monocytes, intermediate monocytes and nonclassical monocytes. The flowcytometric analysis of the three monocyte subpopulations based on expression of CD14 and CD16 suggested that the percentage of the three sub-population does not change significantly after reconditioning (Fig. S1C). Thus, although the reconditioning process tends to enhance the cellular homogeneity of culture by positively selecting monocytes, it does not apparently change the percent abundance of the three monocyte subsets.

After establishing the monocytic origin of RM, the reconditioning induced changes in typical monocytic properties were further analyzed by determining the expression of pan-monocytic marker CD14. The CD14 analysis of monocytes and RM showed a comparative increase in percentage of CD14 positive cells in D6 cultures which in turn can be explained by the increased monocytic homogeneity of D6 cultures (Fig. S1D). As the proportion of non-monocytic cells were high in D0 cultures the percentage of CD14 positive cells was comparatively lower at D0. With an increase in monocytic proportion, the percent of CD14 positive cells also increased at D6 of the culture. However, the notable decrease in CD14 MFI in RM culture denotes the reduced CD14 expression on RM thereby depicting the partial loss of monocytic identity (Fig. S1D).

The analysis of CD16 which is another important marker found on monocytes, showed a reduction in number of CD16 positive cells as well CD16 MFI in RM cultures (Fig. S1E). The reduction in number of CD16 positive cells along with lower expression of CD16 per cell, reaffirms that the process of reconditioning leads to partial loss of monocytic properties in RM. The increase in the expression of CD206 on RM (Fig. S1E) makes them like M2 macrophages.

To further understand the partial differentiation state of RM, the changes in expression of important myeloid lineage markers such as CD115, CD11b, CD163 and CD206 were determined using quantitative PCR (Fig. S1G). The significantly high expression of MCSF receptor (CSF-1R/ CD115) in RM was in coherence with the presence its natural ligand MCSF in the growth factor cocktail. In myeloid cells, the binding of M‐CSF to CSF-1R regulates the generation, differentiation, and functions of macrophages by activating the PI3K/Akt and MAPK pathways. In response to the MCSF induced PI3K/Akt signaling, cells are also known to increase the expression of CD11b (17) and of various cytokines, chemokines, and cell-surface markers related to M2 polarization state (18, 19). Going in accordance with the available literature, the expression of CD11b and CD163 (M2 related marker) was found upregulated in RM which further confirmed the activation of MCSF induced signaling pathway during the process of RM generation. Moreover, the evaluation of CD206 RNA and cell surface expression also indicated the induction of M2 like properties in RM (Fig. S1G).

Thus, the molecular analysis of RM revealed that growth factor cocktail induces partial loss of monocytic markers and MCSF induced upregulation of certain key M2 polarization markers in RM.

In order to determine the reconditioning induced functional changes in cells, the general functional properties of monocytes such as phagocytosis (or efferocytosis), generation of reactive oxygen species and cytokine secretion were evaluated in RM.

To explore the particle uptake potential of RM, cells were incubated with GFP tagged nanoparticles. The confocal imaging of RM treated with GFP-nanoparticle clearly showed the presence of GFP-nanoparticle inside the cellular cytoplasm (Fig. S2A). The nanoparticle uptake by RM suggested its ability of endocytosis, which was shown by the colocalization of nanoparticles with the lysotracker dye. The location of lysotracker dye in the cytoplasm indicates the localization of lysosomes or phagolysosomes (Fig. S2B). Thus, colocalization of GFP nanoparticle within phagolysosomes confirms the phagocytic potential of RM.

*Functional properties of RM*

To further characterize these CD14^lo^ CD16^lo^ RM for their functional properties, their phagocytic ability was evaluated using GFP tagged nanoparticles. The Phagocytic uptake of GFP tagged nanoparticles was first analyzed by confocal microscopy (Fig2A) and was subsequently quantified by flow cytometry (Fig2B). The analysis showed a significant increase in number of cells phagocytosing the nanoparticles. However, the comparison of MFI showed no significant increase in the phenomenon of phagocytosis in RM as compared to monocytes suggesting the unaltered phagocytic potential of RM.

*Real-time qPCR*

RT-qPCR for evaluating the expression of various mRNA transcripts was carried out using GeneSure™ SYBR green qPCR master mix (2X) (Puregene, Genetix) as per manufacturers protocol. Primers used for reaction were designed by the Beacon designer 7 software (Premier Biosoft, USA) and IDT software. The final concentration of the forward and reverse primers used in the reaction mixture was 200nM. The calculation of relative fold gene expression of real time data was done using 2–∆∆Ct method where Ct denotes the cycle threshold (Ct) of samples. The ∆Ct for gene of interest was calculated against Gapdh or β-acting or 18s as house-keeping genes. Calibrator/ reference sample used for calculating relative fold change varied as per the experiments.


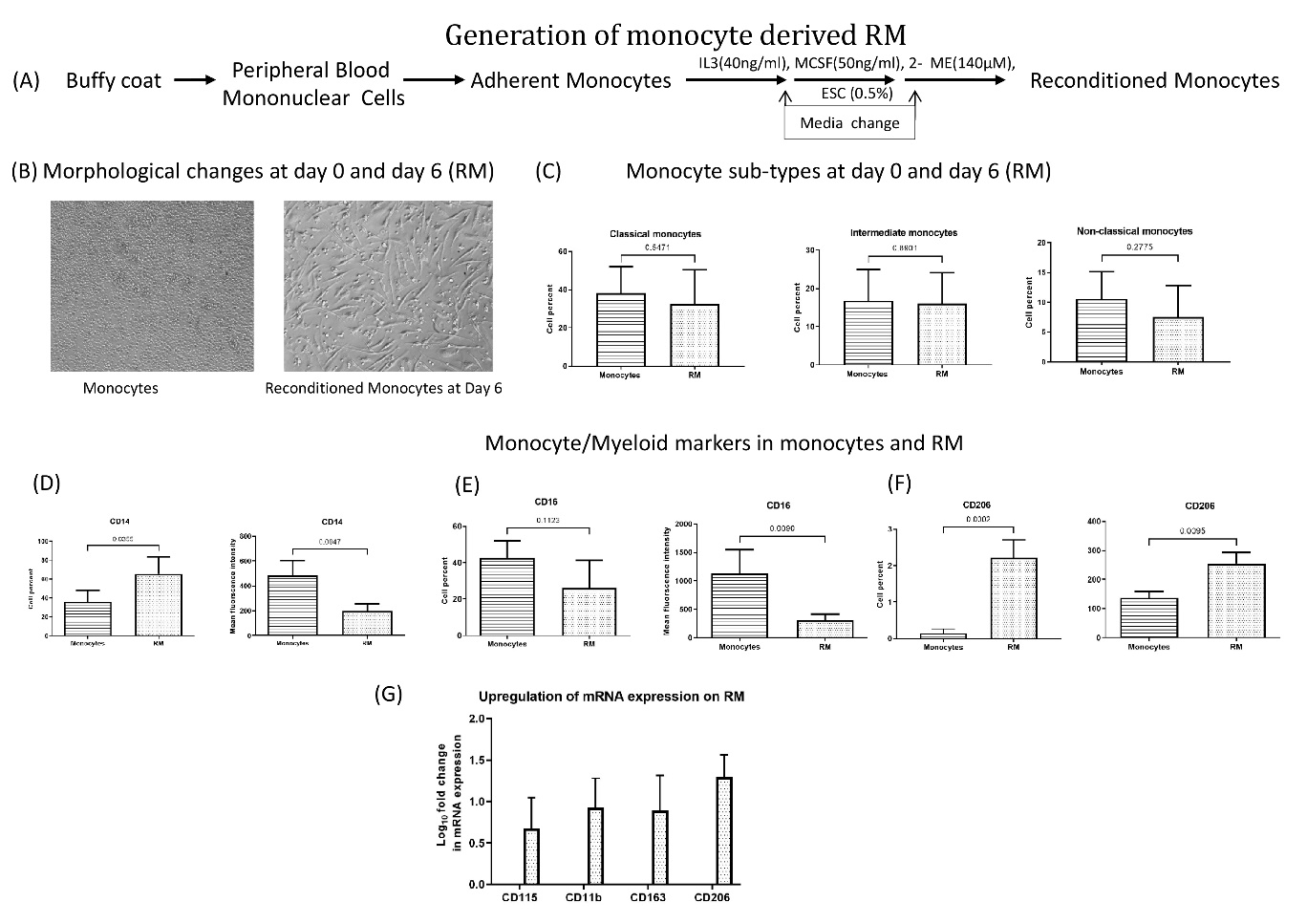


**Figure S1: Phenotypic characterization of Reconditioned Monocytes (RM). (A)** Experimental settings for generation of monocyte derived RM. Monocytes were isolated from buffy coat derived PBMCs based on overnight plastic adherence. Adherent monocytes (D0 population) were subsequently reconditioned to RM (D6 population) using defined media composition, **(B)** Representative Bright-field microscopy pictures of Monocytes and RM showing morphological changes induced in RM after the reconditioning process, **(C)** Bar graphs indicating percentage of Classical, Intermediated and Non-classical monocytes present in monocytes (D0) and RM (D6) based on analysis of CD14 and CD16 expression by flow cytometry (n=6), **(D-H)** Bar graphs indicating differential expression of Monocyte/Myeloid markers in monocytes and RM evaluated by flow cytometry (n=4) **(G)** Bar graphs indicating Differential expression of myeloid lineage cell surface markers in monocytes and RM evaluated by qRT-PCR (n=5). Results are presented as mean with SD.


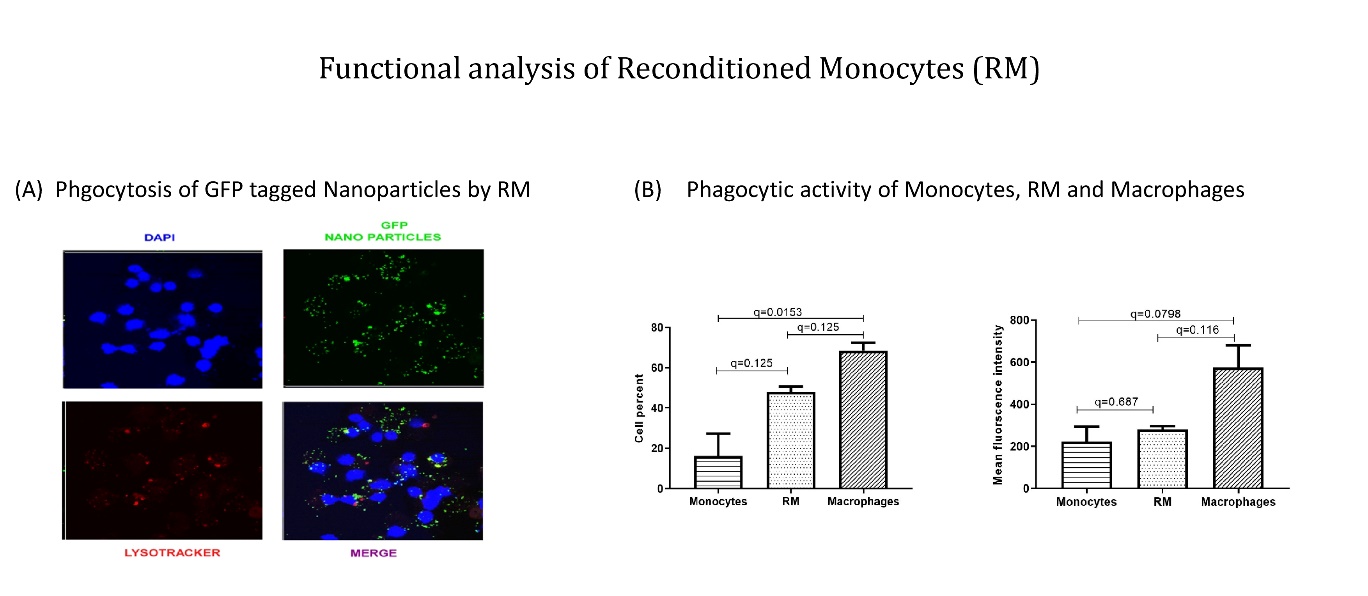


Figure S2: Functional analysis of Reconditioned Monocytes. (A) Representative Confocal microscope image from one experiment showing phgocytosis of GFP tagged Nanoparticles by RM (B) Analysis of Phagocytic activity: Bar graphs indicating phagocytic activity of Monocytes, RM and MCSF induced Mph determined by flow cytometry. Each cell type was incubated for 30 mins with GFP tagged Nanoparticles and percent of cells showing phagocytosis and the extent of phagocytic activity was measured by flow cytometry (n=3). Results are presented as mean with SD.

Section II : Cecal ligation and puncture polymicrobial sepsis model

*Method*

8-10 weeks old male BALB/cj mice weighing between 21-24g, were anesthetized by administration of ketamine-Xylazine mixture intraperitoneally. After 1 to 2 cm long midline laparotomy, the distal end of the cecum was ligated, a single perforation was performed with an 18-gauge needle, and a small amount of stool was taken out. The cecum was then replaced into the peritoneal cavity, and the abdominal incision was sutured 4-0 Chromic suture (Johnson and Johnson Pvt Ltd). Five hundred microliters of 0.9% NaCl was administered sub-cutaneously for fluid resuscitation. To reduce the post-operative pain, animals were given Meloxicam (1mg/ml) (5mg/kg) diluted in saline subcutaneously. Mice were then immediately randomized in different experimental groups. A few combinations of 10^6^ RM, 10^6^ monocytes, antibiotic Ceftriaxone (20mg/Kg) in 150 μl of PBS were administered intraperitoneally, either 4 or 8 hours after CLP procedure. In the control group, only 150 μl PBS was administered.

*Characterization*

In order to validate the reliability of the animal model of sepsis, the overt signs of sepsis like body temperature and weight were determined at early and late time points post-surgery. All the CLP animals showed a progressive weight loss beginning from 6hrs post-surgery time point which is most probably the result of reduced food intake and diarrhea (Fig. S3A). Similarly, a significant decline in the body temperature was observed both at 6 hours and 18 hours post-surgery confirming sepsis induced progressive hypothermia (Fig. S3B).

Studies have reported that among various proinflammatory biomarkers, IL6 is most potent in predicting the disease outcome and can be used as both diagnostic as well as prognostic marker for sepsis (24). MCP1 is another chemoattractant molecule, reported to have a positive correlation with sepsis severity and can be used to predict the sepsis prognosis. Among various anti-inflammatory cytokines, IL10 has been shown critical in sepsis pathophysiology. The levels of IL-6, MCP-1, and IL-10 were significantly higher post-surgery (Fig. S3C).

In healthy liver the key hepatic aminotransferases AST and ALT are generated and stored in hepatocytes whereas in case of liver damage hepatocytes undergo apoptosis thereby releasing these enzymes in circulation. In clinical conditions serum aminotransferase level greater than twice the normal value is considered as a sign of liver failure. Thus, a more than two-fold increase in serum concentration of ALT levels along with a significant rise in AST levels confirmed the liver damage in our animal model (Fig S3D). In line with these results the increased mRNA levels of MCP1 in liver of septic mice indicated increased inflammation in liver (Fig. S3E).

Various reports have shown hypoglycemia as a frequent incident related with liver failure (29) and also as a characteristic of late stages of sepsis. Serum glucose levels measured in current sepsis model clearly indicated hypoglycemia at later stage of the disease (Fig. S3F).

The severity of disease in each group was assessed by comparing their morbidity, mortality, and systemic inflammatory response with the sham control group (Fig. S3G). The comparative analysis of each CLP group with sham control showed that CLP surgery led to a 100% mortality in each group as opposed to the no morbidity and mortality in the sham control, thereby suggesting the success of CLP in inducing sepsis.


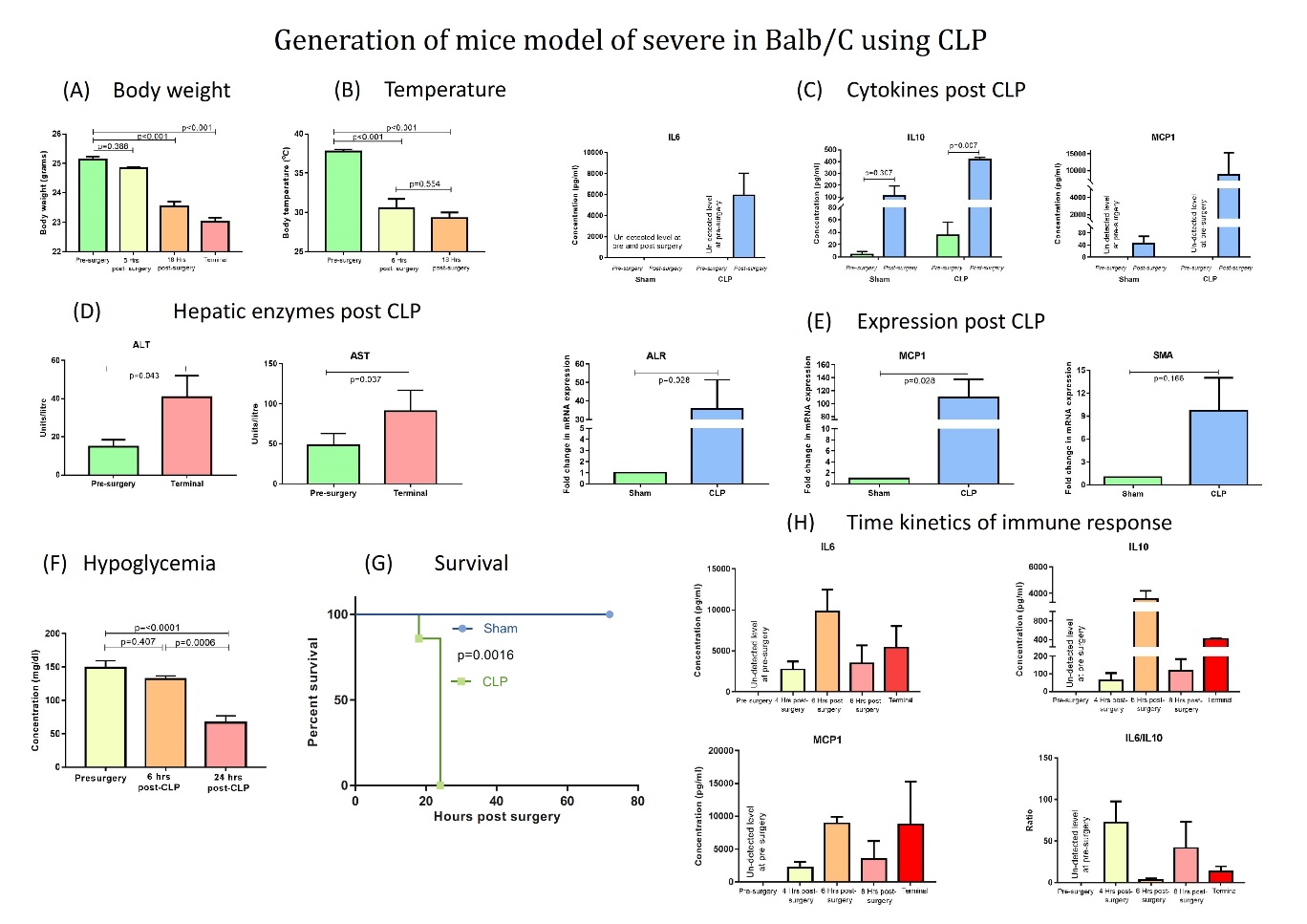


**Figure S3: Generation of mice model of severe in BALB/cj using CLP**

**(A)** Bar graphs representing the progressive reduction in average body weight of animals induced after CLP surgery (n=3)**. (B)** Bar graphs representing the time kinetics of sepsis induced variations in average body temperature (n=3). **(C)** Bar graphs showing mean concentration of serum cytokine in control and CLP animals at 22±2 hrs post-surgery time point (n=3). **(D)** Bar graphs indicating mean concentration of serum ALT and AST levels following CLP-induced sepsis, compared with sham control (n=3). **(E)** Bar graphs indicating relative fold change in mRNA levels of liver inflammation and injury markers following CLP-induced sepsis, compared with sham control (n=4). **(F)** Bar graph depicting serum glucose concentration of CLP animals at various time points(n=5)**. (G)** CLP surgery was done in BALB/cj mice, and mice were monitored for survival. Data for survival of Sham (blue line) and CLP mice (Green) represented as a Kaplan-Meier survival curve (Sham n=5, CLP n=7). **(H)** Bar graphs depicting time kinetics of sepsis induced systemic immune response as indicated by the serum cytokine levels of IL6, IL10 and MCP1(n=3). Results are presented as mean ± SEM.

Section III : Participants inclusion and exclusion for the study

Participation criteria

Inclusion criteria

Exclusion criteria

qSOFA score < 2

Sepsis with clinical sign of infection

Admitted to medicine department of RML hospital

qSOFA score >= 2

Written, informed consent

Age <18 years

Any Malignancy

Chronic inflammatory diseases

Traumatic brain injury

HBV/HCV/HIV infection

Table ST1: Schematic diagram of participants inclusion and exclusion for the study.

Section IV : Creatinine levels in sepsis patients and controls


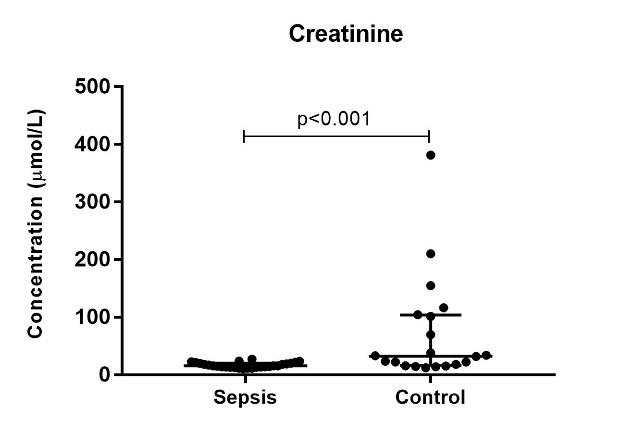


Fig. S4 : Creatinine levels in sepsis patients and controls

Section V


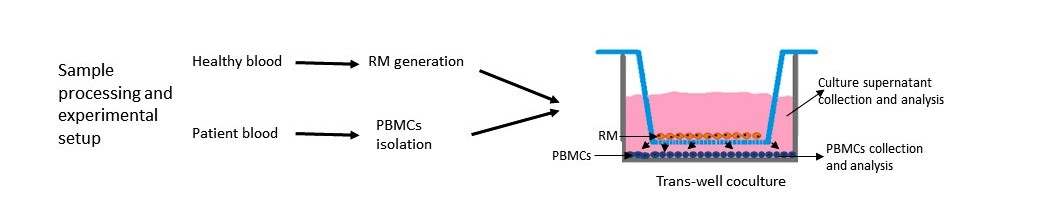


Fig. S5: Schematic flow of sample processing and experimental setup in the RM co-culture with PBMCs isolated from sepsis patients.

Section VI : List of primers:

| **Gene Symbol** | **FORWARD 5'-3'** | **REVERSE 5'-3'** |
| --- | --- | --- |
| **Human primers** | | |
| ***CD115*** | GGAGAGGAACGTGTGTCCAG | GACATAGAGGTGGATGGCGG |
| ***CD163*** | GGATCATGTTTCTTGTCGTGGG | CCACGCGTCAGCCTCATT |
| ***CD206*** | GGAGTATGAAGAGCAAGCC | CATCCGTTTCCACATCAAAT |
| ***Β-actin*** | GTCATCACCATTGGCAAT | TGAAGGTAGTTTCGTGGAT |
| ***Gapdh*** | CAACAGCCTCAAGATCATCAG | GAGTCCTTCCACGATACCAA |
| **Mouse primers** | | |
| ***Mcp1*** | CACTCACCTGCTGCTACTCATT | CTTTGGGGCTACACCTGCT |
| ***Alr*** | GAAGCGGGACATCAAGTTTAGGG | TGAAGGAAAGCCCAGGTGTTG |
| ***Sma*** | ATTCAGGCTGTGCTGTCCCTCTAT | GAGTCACGCCATCTCCAGAGTC |
